# Pre‐anaesthetic risk assessment and management of dogs with myxomatous mitral valve disease: a spectrum of care narrative review

**DOI:** 10.1111/jsap.70119

**Published:** 2026-03-21

**Authors:** I. Levinzon, L. Köster, E. Vettorato, A. H. Estrada

**Affiliations:** ^1^ University of Florida College of Veterinary Medicine Gainesville Florida USA

## Abstract

Myxomatous mitral valve disease, an acquired valvular degeneration, is the most common cardiac disorder in dogs, affecting approximately 10% of dogs in primary care veterinary practice. Dogs with myxomatous mitral valve disease frequently require anaesthesia for routine procedures. The American Society of Anesthesiologists physical status classification system shows increasing mortality with higher grades, but its subjectivity limits risk stratification in dogs with preclinical myxomatous mitral valve disease. Because Stage B1 and B2 dogs can vary widely in disease severity, even within the same American Society of Anesthesiologists category, a single American Society of Anesthesiologists score may not accurately reflect their anaesthetic risk. While a comprehensive echocardiogram remains the gold standard for myxomatous mitral valve disease staging, referral to a veterinary cardiologist is often cost‐prohibitive and might involve long waiting periods. This narrative review proposes a practical framework for pre‐anaesthetic risk assessment and protocol selection in dogs with myxomatous mitral valve disease, integrating accessible diagnostic tools including physical examination, radiographic measurements (vertebral heart size and vertebral left atrial size), cardiac biomarkers (N‐terminal pro‐B‐type natriuretic peptide) and point‐of‐care ultrasound. Recommended anaesthetic protocols are provided for different clinical scenarios ranging from cases with limited diagnostics through grey zone presentations to confirmed cardiomegaly, alongside structured approaches to managing intraoperative complications. This spectrum of care approach recognises that while specialist evaluation remains ideal, practical alternatives can guide safe anaesthetic management for this commonly encountered cardiac condition in companion dogs.

## INTRODUCTION

Myxomatous mitral valve disease (MMVD) is the most common cardiac disorder in dogs, affecting approximately 10% of dogs seen in primary care veterinary practices (Keene et al., 2019). The disease involves progressive degeneration of the mitral valve apparatus, leading to mitral regurgitation (MR) and subsequent cardiac remodelling (Oyama & Levy, 2010; Perkowski & Oyama, 2024), creating significant challenges for safe anaesthetic management (Borgarelli et al., 2008).

The American Society of Anesthesiologists (ASA) physical status classification system demonstrates escalating peri‐anaesthetic mortality rates, ranging from 0.08% in ASA 1 dogs to 15.73% in ASA 5 dogs (Redondo et al., 2024). Preclinical MMVD, as defined by the ACVIM consensus (Keene et al., 2019), includes dogs in stage B1, characterised by structural valvular disease without echocardiographic evidence of cardiac remodelling and not requiring cardiologic treatment, and stage B2, in which cardiac remodelling is present and severe enough to recommend treatment with pimobendan (further details on the echocardiographic cut‐offs used to define this stage are provided below). In the authors' opinion, dogs with stage B1 MMVD are frequently classified as ASA 2, while those in stage B2 are often considered ASA 3 (Portier & Ida, 2018). This distinction implicitly acknowledges the increased peri‐anaesthetic risk associated with stage B2 disease, including a potentially higher risk of adverse outcomes in the first 24 hours following anaesthesia (Redondo et al., 2024). Consequently, anaesthetic planning for dogs with stage B2 MMVD requires greater caution and individualised consideration. However, considerable heterogeneity exists within stage B2 MMVD, as some dogs barely meet treatment thresholds while others approach congestive heart failure. For example, chamber size and MR fraction considered severe would be equivalent to Stage C in terms of anaesthesia risk despite the dog remaining preclinical. A single ASA classification may not adequately capture this variation, as the ASA system does not account for the spectrum of disease severity within preclinical MMVD and lacks cardiovascular specificity. As a result, dogs with markedly different cardiac risk profiles may receive the same ASA classification. This limitation underscores the need for a more nuanced approach to peri‐anaesthetic risk assessment in dogs with preclinical MMVD and highlights the clinical relevance of this narrative review.

Comprehensive cardiac evaluations are often linked with additional financial burdens and may result in longer waiting periods. While specialist echocardiography remains the gold standard for MMVD staging, the 2019 ACVIM consensus provides guidance for staging using murmur grading and thoracic radiographs when echocardiography is unavailable (Keene et al., 2019). Other validated techniques include cardiac biomarkers [N‐terminal pro‐B‐type natriuretic peptide (NT‐proBNP)] and point‐of‐care ultrasound (POCUS) (Dickson et al., 2022; Malcolm et al., 2018; Oyama & Singletary, 2010; Vezzosi et al., 2021).

This review aimed to describe accessible pre‐anaesthetic diagnostic tools for dogs with MMVD, propose a practical risk assessment algorithm highlighting the limitations of the ASA classification and provide protocol recommendations based on clinical presentation and available diagnostic resources.

## LITERATURE SEARCH METHODS

A comprehensive literature search was conducted using PubMed/MEDLINE and CAB Abstracts. Search strategies combined core terms (canine OR dog) AND (MMVD OR myxomatous mitral valve disease) with specific modalities: anaesthesia/anaesthetic management, physical examination/auscultation, radiographic measurements (VHS, VLAS), cardiac biomarkers (NT‐proBNP), POCUS and artificial intelligence tools. Additional searches targeted anaesthetic drugs, haemodynamic management and perioperative complications in dogs with cardiac disease. Articles were also identified through reference lists of key publications and expert recommendations. Studies were included if they addressed MMVD staging, cardiac diagnostic tools (radiography, biomarkers, POCUS and artificial intelligence), anaesthetic protocols or perioperative complications in dogs. Studies focussing on surgical correction of MMVD were excluded. Studies were selected based on clinical relevance to pre‐anaesthetic assessment and anaesthetic management of dogs with MMVD, with emphasis on diagnostic tools available in general practice. The quality of evidence varied from prospective randomised trials to retrospective case series.

## PRE‐ANAESTHETIC ASSESSMENT TOOLS

### Physical examination: auscultation, murmur grading and clinical signs

Initial assessment involves evaluating murmur characteristics (point of maximal intensity, radiation and grade/intensity), physical examination findings (mucous membrane colour, femoral pulse quality, heart rate and rhythm, and respiratory rate) and signalment (Côté et al., 2015).

Murmur intensity is traditionally graded using a 6‐level system: Grade I/VI (very faint), Grade II/VI (soft), Grade III/VI (moderate intensity), Grade IV/VI (loud), Grade V/VI (very loud) and Grade VI/VI (audible with stethoscope off chest wall, with precordial thrill) (Levine, 1933; Ljungvall et al., 2009). A simplified 4‐level descriptive scheme has been validated and may improve inter‐observer agreement, categorising murmurs as “soft” (Grades I and II/VI), “moderate” (Grade III/VI), “loud” (Grade IV/VI) and “palpable” (Grades V and VI/VI with thrill) (Caivano et al., 2018; Ljungvall et al., 2014).

In small‐breed dogs, such as Cavalier King Charles Spaniels and dachshunds (Keene et al., 2019), with left apical systolic murmurs, MMVD is the most common cause, though differential diagnoses include mitral valve dysplasia, endocarditis, severe anaemia and breed‐associated variation (Ljungvall et al., 2009). Concomitant tricuspid regurgitation occurs in approximately 30% of dogs with MMVD, which may result in additional right‐sided murmurs (Keene et al., 2019). While murmur intensity correlates with MR severity (Häggström et al., 1995; Ljungvall et al., 2014), other factors can also influence murmur intensity, regardless of disease severity. These include respiratory sinus arrhythmia, reactive or situational hypertension, or eccentricity of the regurgitant jet relative to the dog's body conformation (Aker et al., 2025; Keren et al., 1989; Pedersen et al., 1999; Reimann et al., 2014; Wooley, 1978). Clinical signs suggesting cardiac decompensation include exercise intolerance, tachypnoea, dyspnoea, collapse, tachycardia and arrhythmias (Boswood et al., 2016). Exercise intolerance has limited utility as a screening criterion, as preclinical dogs with moderate or louder murmurs rarely demonstrate reduced exercise capacity at baseline (Boswood et al., 2016). Cough should be interpreted cautiously, as it may be associated with concurrent airway disease rather than cardiac decompensation in this population (Ferasin & Linney, 2019; Rishniw et al., 2025).

The 2019 ACVIM consensus recommends cardiac workup for murmurs Grade III/VI or louder (Keene et al., 2019), but the anaesthetic risk profile for dogs with soft murmurs (Grades I and II/VI) remains less well‐defined. For pre‐anaesthetic assessment, in the authors' opinion, additional cardiac diagnostics should be considered for all dogs with murmurs. However, when not feasible, dogs with Grades I and II/VI murmurs may represent lower anaesthetic risk if they exhibit absence of respiratory signs and resting respiratory rates consistently below 25 to 30 breaths per minute (Boswood et al., 2020; Rishniw et al., 2012). Red flags mandating cardiac evaluation include the following: Grade III/VI or louder murmurs, signs of decompensation (exercise intolerance, respiratory distress and collapse), physical examination abnormalities (crackles and arrhythmias) or murmurs radiating beyond the point of maximal intensity (López‐Alvarez et al., 2015).

### Radiographic methods: vertebral heart size and vertebral left atrial size

Radiographic assessment plays an important role in staging MMVD when echocardiography is unavailable. The 2019 ACVIM Consensus Statement recognises radiographic signs of cardiomegaly as a potential substitute for echocardiographic assessment (Keene et al., 2019). Two quantitative measurements are widely used: Vertebral Heart Size (VHS) and Vertebral Left Atrial Size (VLAS).

### Vertebral heart size

The VHS provides a generalised assessment of cardiac size, calculated by summing the perpendicular maximum cardiac silhouette length and width normalised to vertebral body units beginning at T4 (Figs 2 and 4) (Buchanan & Bücheler, 1995). Normal dogs have a mean VHS of 9.7 vertebrae (range 9.2 to 10.2), while dogs with MMVD demonstrate progressively elevated values with advancing disease stages (An et al., 2023; Buchanan & Bücheler, 1995). The ACVIM consensus lists breed‐adjusted VHS >10.5 as one of the Stage B2 criteria when echocardiography is unavailable (Keene et al., 2019). The guidelines acknowledge that definitive radiographic criteria for Stage B2 identification are not currently available. In the absence of echocardiography, clear radiographic cardiomegaly evidence, such as a “general breed” VHS ≥11.5 or a comparable “breed‐adjusted” VHS where breed‐specific normal values are available, can substitute for quantitative echocardiography to identify Stage B2 (Keene et al., 2019). To date, VHS values have been published for more than 20 canine breeds; however, the majority of these studies have limited sample sizes, often well below the 120 individuals considered necessary for establishing statistically reliable reference ranges, potentially limiting the clinical applicability of VHS interpretation in some cases (Bagardi et al., 2022; Battinelli et al., 2024; Friedrichs et al., 2012). The authors' opinion is that breed‐specific VHS values are an appropriate clinical tool for decision‐making when classifying MMVD for anaesthetic or treatment purposes.

### Vertebral left atrial size

Vertebral left atrial size quantifies left atrial enlargement similar to a VHS on a right lateral thoracic radiograph, from the ventral carina to the dorsal intersection of the cardiac silhouette and caudal vena cava, transposed to T4 (Figs 3 and 5) (Malcolm et al., 2018). The VLAS correlates with echocardiographic left atrial measurements and may be more specific than VHS for predicting left‐sided enlargement in small breeds (Duler et al., 2021; Malcolm et al., 2018). The ACVIM consensus recommends VLAS ≥3.0 as the threshold for Stage B2 identification in the absence of echocardiography (Keene et al., 2019; Malcolm et al., 2018). Some studies suggest this cut‐off may be too high, although it is apparent that a proposed cut‐off below this (VLAS 2.3 to 2.6) has poor performance, with reports of 72% to 95% sensitivity and 78% to 84% specificity for detecting cardiac remodelling (Levicar et al., 2022; Malcolm et al., 2018; Wesselowski et al., 2023). The aim of the ACVIM consensus is clearly to ensure better specificity before prescribing pimobendan; however, it does not consider the implications in terms of ASA status, where being more careful would be the goal, and perhaps a superior sensitivity and therefore lower cut‐off may be optimal at the expense of false positives.

## PRACTICAL APPLICATION FOR ANAESTHETIC RISK ASSESSMENT

For pre‐anaesthetic evaluation, radiographic measurements help stratify risk when integrated with physical examination findings. Dogs with VHS <10.5 and VLAS <2.3 typically correspond to Stage B1. Dogs with VHS 10.5 to 11.5 or VLAS 2.3 to 3.0 represent a diagnostic grey zone between Stage B1 and B2. Gold standard assessment with POCUS, NT‐proBNP or cardiology consultation could be pursued to clarify disease stage before anaesthesia. However, the authors' opinion is that good clinical practice when additional diagnostics are either not possible or not feasible and anaesthesia must proceed is to err on the side of caution for patients in this diagnostic grey zone. Anaesthetic risk allocation should be higher (as below) when these thresholds are exceeded (VHS≥10.5 and VLAS ≥2.3).

Dogs with VHS ≥11.5 or VLAS ≥3.0 are expected to be at least Stage B2 and require enhanced anaesthetic protocols. The presence of radiographic pulmonary oedema indicates Stage C disease, which represents a contraindication to elective anaesthesia until cardiac stabilisation occurs.

Important limitations include patient positioning variability, breed‐specific variations in normal values and the challenge of two‐dimensional assessment of three‐dimensional cardiac structures (Hansson et al., 2005; Malcolm et al., 2018).

### Cardiac biomarkers – NT‐proBNP testing

B‐type natriuretic peptide (BNP) is a cardiac hormone released by myocardial cells in response to increased cardiac stretch. NT‐proBNP, the inactive fragment of proBNP, has become the preferred marker in veterinary medicine due to its longer half‐life and greater stability during sample processing (Hall, 2004). NT‐proBNP demonstrates utility in differentiating active congestive heart failure from primary respiratory disease and in assessing disease severity (Oyama & Singletary, 2010).

In MMVD, NT‐proBNP concentrations increase with disease progression, with Stage C dogs showing higher levels than Stage B2 and healthy controls (Ruaux et al., 2015; Winter, Saunders, Gordon, Miller, et al., 2017). However, NT‐proBNP concentration overlap between stages limits definitive categorization in individual dogs.

Available assays demonstrate strong diagnostic performance for identifying congestive heart failure (Stage C MMVD), with reported sensitivities of 90% to 100% and specificities of 81% to 85% (Riengvirodkij & Sakcamduang, 2021). However, as a stand‐alone diagnostic tool for staging MMVD, these assays show limited ability to discriminate early disease, with AUC values of 0.77 to 0.86. Diagnostic accuracy improves substantially when assay results are combined with other clinical variables – including murmur intensity, VHS and physical examination findings – with reported AUCs of 0.90 to 0.97 (Wesselowski et al., 2023; Wilshaw et al., 2021).

Optimal cut‐off values vary substantially across assay platforms, making platform‐specific interpretation essential. For commonly used IDEXX Cardiopet proBNP assays, reported cut‐offs for Stage B2 identification range from 1100 to 1453 pmol/L (Ogawa et al., 2021; Wesselowski et al., 2023; Wilshaw et al., 2021). For congestive heart failure detection, reported cut‐offs range from 787 to 1772 pmol/L across various assay platforms (Chanmongkolpanit et al., 2024; Ogawa et al., 2021; Wolf et al., 2013). Several factors can influence NT‐proBNP levels, including renal disease (particularly azotaemia with creatinine >1.9 mg/dL), systemic hypertension and breed variations (Harr et al., 2022; Raffan et al., 2009; Schmidt et al., 2009). For example, Doberman Pinschers have a lower optimised decision threshold of 457 pmol/L for identifying occult dilated cardiomyopathy, whereas healthy Greyhounds have a notably higher mean concentration of 946 pmol/L (Harr et al., 2022). Physical activity can also increase NT‐proBNP concentrations, both after prolonged strenuous exercise (Hunt et al., 2018) and short‐duration high‐intensity sessions (Ivasovic et al., 2023), sometimes exceeding reference ranges. Pimobendan may lower NT‐proBNP values by reducing cardiac wall stress (Iwanuk et al., 2019). Additionally, NT‐proBNP exhibits high biologic variability; for dogs with MMVD, a minimum increase of approximately 58% between successive measurements is required to signify true pathological change (Winter, Saunders, Gordon, Buch, et al., 2017).

Point‐of‐care immunoassays provide rapid results (de Lima & da Silveira Ferreira, 2017). Proper sample handling is critical: immediate centrifugation and analysis within two hours, or freezing for longer delays (Chanmongkolpanit et al., 2024).

## PRACTICAL APPLICATION FOR ANAESTHETIC RISK ASSESSMENT

NT‐proBNP demonstrates good diagnostic performance for distinguishing dogs with congestive heart failure (Stage C) from asymptomatic dogs (Stages B1 and B2), with sensitivity of 90% to 100% (Riengvirodkij & Sakcamduang, 2021). Elevated levels suggesting Stage C disease (typically >1500 to 1800 pmol/L for IDEXX assays) warrant cardiac stabilisation and consideration of delaying elective procedures. To the best of the authors' knowledge, there is currently no evidence supporting the use of NT‐proBNP for peri‐anaesthetic risk stratification in dogs with preclinical MMVD. However, as NT‐proBNP reflects myocardial wall stress and neurohormonal activation, it may have potential value in improving pre‐operative risk stratification within the heterogeneous population of preclinical MMVD dogs, particularly those in stage B2. In dogs with stable Stage C, targeting NT‐proBNP below 1500 pmol/L through dose adjustment may indicate a more favourable anaesthetic candidate (Hezzell et al., 2018).

### Point‐of‐care ultrasound

Point‐of‐care ultrasound in veterinary cardiology involves echocardiographic imaging performed and interpreted by the treating clinician for immediate clinical integration (Kirkpatrick et al., 2024). The American Society of Echocardiography distinguishes between basic cardiac POCUS (B‐mode imaging with optional M‐mode and colour Doppler) and advanced cardiac POCUS (incorporating spectral Doppler for quantitative haemodynamic measurements) (Kirkpatrick et al., 2024).

For MMVD staging, key echocardiographic measurements are the normalised left ventricular internal diameter in diastole (LVIDdN) and left atrial‐to‐aortic ratio (LA:Ao). According to ACVIM guidelines, dogs meet Stage B2 criteria when LVIDdN ≥1.7 and LA:Ao ≥1.6 (Keene et al., 2019). Studies demonstrate that non‐specialist practitioners can achieve substantial agreement with board‐certified cardiologists in MMVD staging following targeted training (Beber et al., 2018; Dickson et al., 2022; Wesselowski et al., 2022). Training programmes of 2 to 3 hours can achieve competency for Stage B1 *versus* B2 differentiation, though underestimation of measurements such as LVIDdN by non‐specialists can occur, potentially leading to misclassification of Stage B2 dogs as Stage B1 (Dickson et al., 2022; Levinzon et al., 2024).

The American Society of Echocardiography emphasises that training must match examination complexity, with both basic and advanced cardiac POCUS requiring formal training programmes for patient safety (Kirkpatrick et al., 2024). Cardiac POCUS examinations should be documented with structured reports clearly identifying the examination type performed.

## PRACTICAL APPLICATION FOR ANAESTHETIC RISK ASSESSMENT

In the pre‐anaesthetic setting, cardiac POCUS should be utilised as a qualitative tool for interpretation of cardiac dimensions and function (DeFrancesco & Ward, 2021), rather than for quantitative differentiation of MMVD stages, as linear measurements such as LA:Ao are subject to significant inter‐operator variability (Hsue & Visser, 2020; Safian et al., 2022). Its clinical role is most established in emergency triage to identify overt abnormalities such as B‐lines indicative of pulmonary oedema or pericardial effusion (Ward & DeFrancesco, 2023). For less experienced operators, the anteroposterior maximum left atrial diameter from a right parasternal long‐axis four‐chamber view may be more reproducible (Safian et al., 2022). As with other diagnostic modalities, POCUS measurements should be interpreted alongside clinical findings and patient history rather than as stand‐alone assessments. When available, POCUS can help clarify disease stage in dogs with borderline radiographic measurements or discordant diagnostic findings.

### Artificial intelligence‐based tools

Artificial intelligence tools are increasingly used in veterinary cardiology to reduce measurement variability and improve efficiency (Vrudhula et al., 2024). AI algorithms have been developed for automated VHS measurement, MMVD stage classification from thoracic radiographs and echocardiographic chamber measurements (Banzato et al., 2021; Burti et al., 2020; Solomon et al., 2023; Stowell et al., 2024; Valente et al., 2023). An AI algorithm for automated VHS measurement demonstrated performance comparable to board‐certified veterinary cardiologists, with a mean difference of 0.09 vertebrae between algorithm and manual measurement (Solomon et al., 2023).

Despite proven effectiveness, widespread clinical implementation remains limited, with commercial systems reaching only approximately 5% of US veterinary practices (Ndiaye et al., 2025). AI‐based tools for VHS measurement and MMVD staging may serve as adjuncts to clinical assessment when available, though results should be interpreted alongside other clinical findings rather than used as stand‐alone diagnostic methods.

Table 1 summarises the relative costs, time requirements and clinical utility of various diagnostic approaches for MMVD assessment in general practice.

**Table 1:** Cost‐effective comparison of diagnostic approaches for MMVD assessment (costs in USD, United States)

Assessment method	Equipment cost	Per‐case cost	Time required	Clinical value
Physical examination	$50 to 200 (stethoscope)	$90 to 100	5 to 10 minutes	Baseline screening
Radiographs + VHS/VLAS	$25,000 to 150,000	$225 to 235	15 to 20 minutes	Structural assessment
NT‐proBNP (POC)	$2000 to 4500	$95 to 120	15 minutes	Functional assessment
NT‐proBNP (reference)	None	$95 to 120	24 to 48 hours	Functional assessment
Cardiac POCUS	$2700 to 10,000 (*e.g*. handheld device − traditional systems) + training*	$150	10 to 15 minutes	Comprehensive staging
AI‐assisted tools	Variable	$225 to 235	15 to 20 minutes	Automated interpretation

Costs represent approximate values in United States dollars (USD) based on pricing in the United States, 2024 to 2025

AI Artificial intelligence, NT‐proBNP N‐terminal pro‐B‐type natriuretic peptide, POC Point‐of‐care, POCUS Point‐of‐care ultrasound, VHS Vertebral heart size, VLAS Vertebral left atrial size

* Equipment examples include handheld device and traditional ultrasound systems

### Integrated assessment and clinical decision‐making

**FIG 1:**
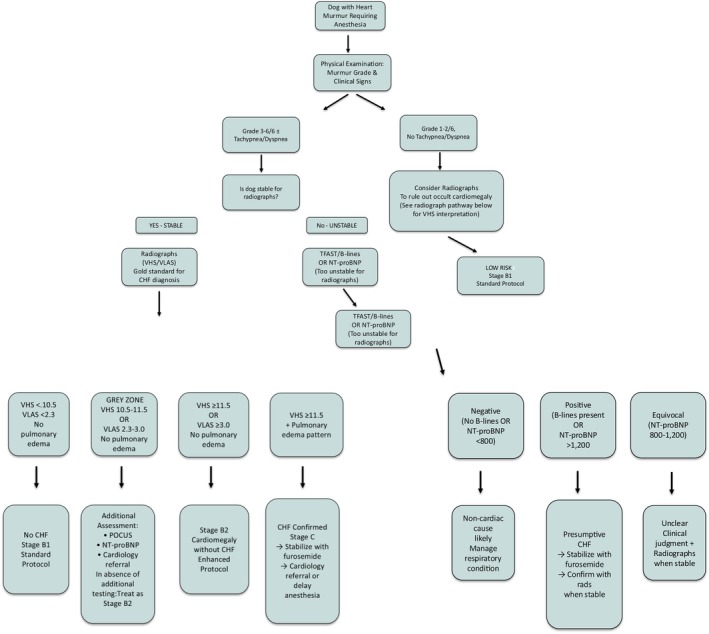
Pre‐anaesthetic risk assessment algorithm for dogs with MMVD. The flowchart guides integration of physical examination findings and radiographic measurements (VHS/VLAS) to stratify anaesthetic risk. Grey zone values (VHS 10.5 to 11.5 or VLAS 2.3 to 3.0) indicate need for additional assessment with POCUS, NT‐proBNP or cardiology referral when feasible. Pulmonary oedema indicates Stage C disease requiring stabilisation before elective anaesthesia. Practitioners should select diagnostic approaches based on available resources and clinical urgency. The recommendations presented represent the authors' clinical approach and do not derive from controlled studies; individual protocols may vary among practitioners.

Figure 1 illustrates a suggested algorithm that can be implemented in clinical practice for integrating physical examination findings and radiographic measurements to guide pre‐anaesthetic risk assessment in dogs with MMVD.

### Anaesthetic considerations in preclinical MMVD: current data and outcome

Pre‐anaesthetic risk assessment is paramount for decreasing the risk of perioperative complications and improving outcomes. ASA score classifies the pre‐anaesthetic physical status of an animal: 1 = healthy with no disease; 2 = healthy with a mild systemic disease without substantive functional limitation; 3 = presence of a severe systemic disease with substantive functional limitation; 4 = a patient with a systemic disease that could die within 24 hours; 5 = a moribund patient unlikely to survive 24 hours with or without surgery (Portier & Ida, 2018). While the ASA score does not directly quantify the anaesthetic risk, as the latter is multifactorial, dogs with an ASA score ≥3 have an increased risk of anaesthetic mortality until 24 to 72 hours after anaesthesia (Portier & Ida, 2018; Redondo et al., 2024). Nevertheless, ASA score appears too broad for stratifying the risk for all MMVD stages, especially considering that the MMVD severity varies considerably even within individual ACVIM stages. Therefore, the clinician should familiarise themselves with the main anaesthetic considerations and tailor the anaesthetic protocol depending on the pre‐anaesthetic clinical findings and the severity of the MMVD.

### Anaesthetic considerations for MMVD

Anaesthetic management for dogs with MMVD presents unique challenges, yet current evidence suggests that with appropriate modifications and monitoring, anaesthetic risk might not be significantly increased for subclinical cases. One retrospective study indicated that dogs with heart disease, including those with MMVD classified as ACVIM Stage B2 or higher, undergoing routine dental procedures at a referral centre experienced no more anaesthetic complications or mortality than healthy dogs (Carter et al., 2017).

The primary goal of anaesthesia in patients with MR is to maintain forward aortic flow while minimising regurgitant flow and development of pulmonary oedema. Most patients with MR benefit from afterload reduction. By reducing afterload, left ventricular systolic pressures required for ejection decrease, optimising forward flow and decreasing the pressure gradient from the left ventricle to the left atrium during systole (Perkowski & Oyama, 2024). Dogs with chronic, compensated MR are sensitive to changes in ventricular loading conditions, and preload should generally be maintained during anaesthesia (Crosland et al., 2021). Excessive fluid administration should be avoided to prevent ventricular distension. Maintenance fluid rates of 2 to 5 mL/kg/hour are generally recommended (Cooke & Snyder, 1998; Pardo et al., 2024). Heart rates should be maintained at normal or slightly above normal rates to help maintain smaller left ventricular volumes and minimise MR secondary to annular dilation (Crosland et al., 2021; Seymour et al., 2008). Bradycardia increases the duration of systolic contraction and the time available for regurgitant flow (Perkowski & Oyama, 2024).

Patients receiving cardiac medications should generally continue them through anaesthesia, particularly diuretics, calcium channel blockers, antiarrhythmics and inotropes. Oral pimobendan should be administered on the day of anaesthesia, as it is considered essential for MMVD management in both Stage B2 and Stage C (Boswood et al., 2016). The role of injectable pimobendan in the anaesthetic setting remains unclear, though one study demonstrated that intravenous administration provides a rapid inotropic effect and reduces left ventricular end‐diastolic pressure (Hori et al., 2019). Hypotension may be more pronounced in patients receiving ACE inhibitors; some anaesthesiologists prefer to withhold enalapril until after recovery (Coleman et al., 2016). This approach should not be considered deleterious for the management of underlying MMVD, as recent studies have demonstrated that ACE inhibitors do not significantly change survival in dogs with MMVD at either Stage B2 or Stage C (Borgarelli et al., 2020; Romito, Ghilardi, et al., 2025; Wess et al., 2020). Inotropic support (*e.g*. dobutamine) may be necessary for advanced MMVD Stage B2, see case example (Goya et al., 2018).

Comprehensive monitoring is essential and has been shown to reduce anaesthesia‐related mortality (Brodbelt et al., 2008). A dedicated clinician or well‐trained technician should repeatedly assess depth of anaesthesia, pulse rate and quality, capillary refill time and body temperature. A pulse oximeter (SpO_2_), noninvasive blood pressure, electrocardiography and capnography should be continuously monitored if these are available (Bailey et al., 2025; Crosland et al., 2021).

### Stage B1 – low‐risk anaesthetic approach

#### Clinical example

**FIG 2:**
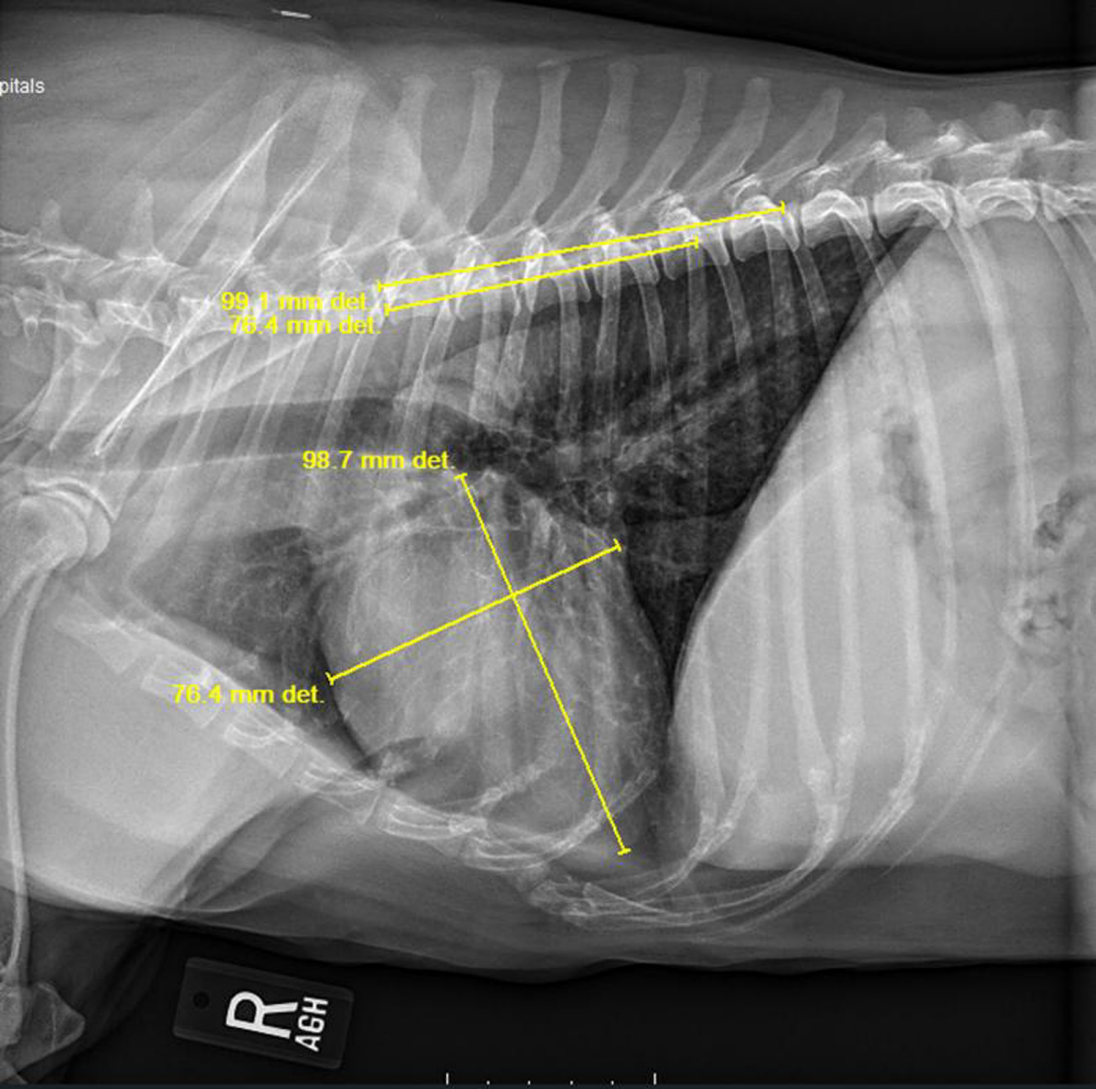
Right lateral thoracic radiograph demonstrating vertebral heart size (VHS) measurement of 10.3 vertebrae in a dog.

**FIG 3:**
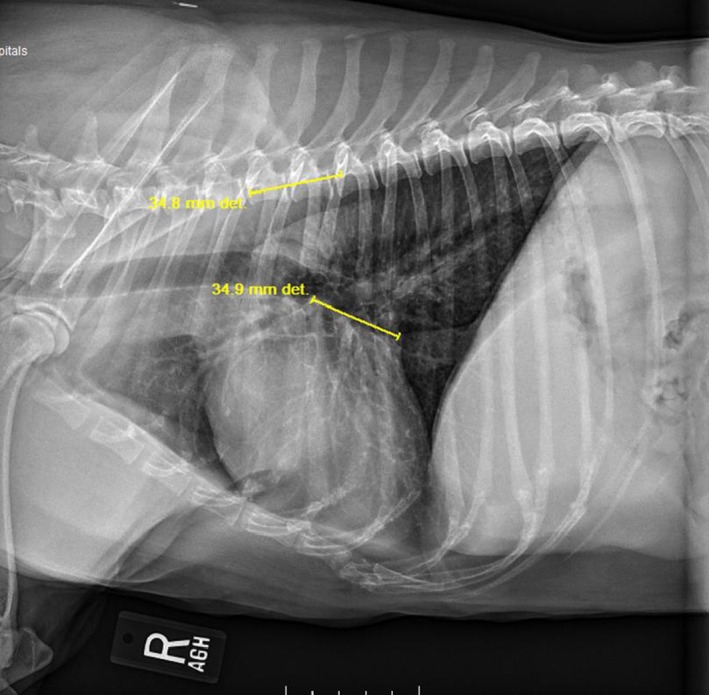
Right lateral thoracic radiograph demonstrating vertebral left atrial size (VLAS) measurement of 2.2 vertebrae in a dog.

A 10‐year‐old mixed‐breed dog (8 kg) with a Grade II/VI left apical systolic murmur requires anaesthesia for cystotomy. Radiographs reveal VHS 10.3 (Fig 2) and VLAS 2.2 (Fig 3), consistent with Stage B1.

Dogs with Stage B1 MMVD have mild MR without cardiac remodelling, typically presenting with soft murmurs (Grades I and II/VI), normal exercise tolerance and diagnostic findings supporting early disease (VHS <10.5, VLAS <2.3, normal NT‐proBNP when assessed). These patients can proceed with standard anaesthetic protocols with minor modifications to optimise cardiovascular stability.

#### Premedication

An opioid–acepromazine combination is appropriate for anxious dogs, as the peripheral α₁‐receptor antagonism induced by acepromazine reduces afterload and improves forward cardiac output (Pypendop & Verstegen, 1998; Rangel et al., 2020). In the authors' opinion, low doses of α₂‐agonists (*e.g*. dexmedetomidine 0.5 to 1 μg/kg iv or 2 to 3 μg/kg im) could also safely used to sedate nervous dogs with stage B1 MMVD. Co‐administration of vatinoxan, a peripheral α₂‐antagonist, with medetomidine reduces the negative impact on afterload and heart rate compared with medetomidine alone, potentially expanding the safe use of α₂‐agonists in dogs with MR (Sarotti et al., 2024; Välimäki et al., 2024).

#### Induction

Propofol (2 to 6 mg/kg iv) or alfaxalone (1 to 3 mg/kg iv) has similar cardiovascular properties and is both an appropriate choice (Maney et al., 2013; Sarotti et al., 2024). Co‐induction with fentanyl and/or midazolam reduces total dose requirements (Okushima et al., 2015; Sánchez et al., 2013). Ketamine combined with benzodiazepine or propofol can also be used (Martinez‐Taboada & Leece, 2014).

#### Maintenance

Isoflurane or sevoflurane can be safely used. To reduce the volatile anaesthetic requirement and cardiovascular depression, regional anaesthesia or partial intravenous anaesthesia (PIVA) can be used. The latter represent the infusion of additional analgesics (*i.e*. fentanyl 5 to 10 μg/kg/hour, ketamine 0.3 to 0.6 mg/kg/hour or lidocaine 1 to 2 mg/kg/hour) to reduce the minimum alveolar concentration of the volatile anaesthetic (Perkowski & Oyama, 2024). An antimuscarinic agent (*e.g*. atropine or glycopyrrolate) should be readily available if bradycardia develops (Perkowski & Oyama, 2024).

#### Recovery

A calm environment is essential. Light sedation should be provided if needed (Crosland et al., 2021). Nonsteroidal anti‐inflammatory (NSAIDs) drugs are not contraindicated in normotensive, normovolaemic patients. The risk of overload from fluid therapy is low. An initial rate of 5 mL/kg/hour is suggested (Boysen & Gommeren, 2021; Pardo et al., 2024).

### Stage B2 – confirmed cardiomegaly anaesthetic approach

#### Clinical example

**FIG 4:**
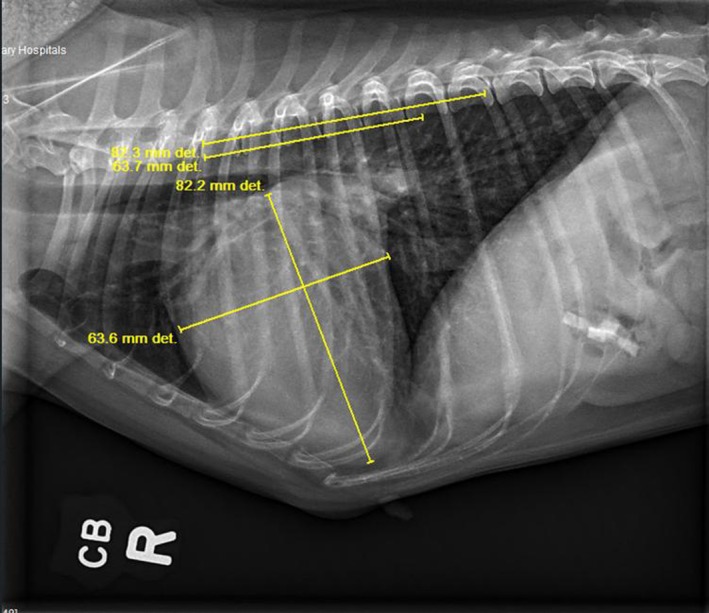
Right lateral thoracic radiograph demonstrating vertebral heart size (VHS) measurement of 12.4 vertebrae in a dog.

**FIG 5:**
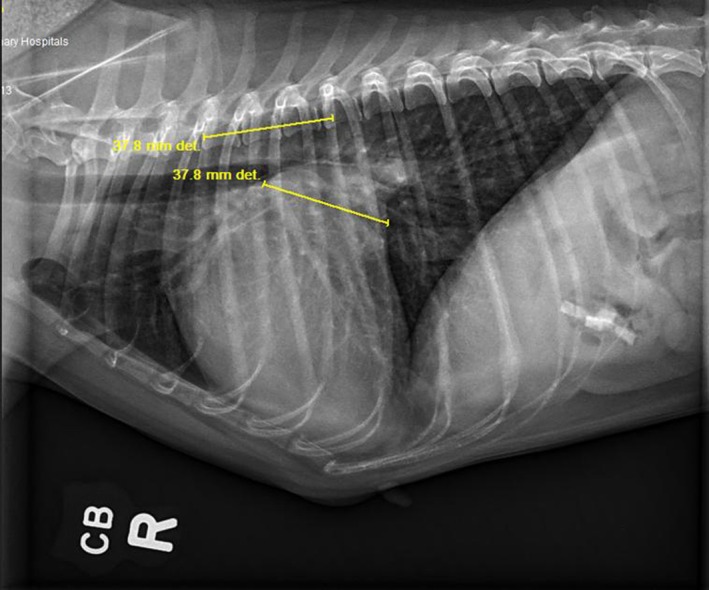
Right lateral thoracic radiograph demonstrating vertebral left atrial size (VLAS) measurement of 3.2 vertebrae in a dog.

An 11‐year‐old Maltese with a Grade IV/VI left apical systolic murmur requires anaesthesia for cutaneous mass removal. VHS measures 12.4 (Fig 4) and VLAS 3.2 (Fig 5), consistent with Stage B2. The dog receives pimobendan 0.25 mg/kg twice‐daily.

Dogs with Stage B2 MMVD, as per the EPIC criteria, have sufficient cardiac remodelling that they would likely benefit from pimobendan in terms of delayed onset of CHF (Boswood et al., 2016). These patients require tailored anaesthetic protocols with enhanced monitoring and readiness for haemodynamic support.

#### Premedication

Opioids (butorphanol, methadone and buprenorphine) provide mild sedation with minimal cardiovascular effects (Perkowski & Oyama, 2024). Maropitant prevents emesis when using hydromorphone or morphine (Hay Kraus, 2014). For dogs needing deeper sedation, opioid–acepromazine combinations remain appropriate with lower acepromazine doses than Stage B1. Alpha‐2 agonists are contraindicated as their α‐mediated vasoconstriction increases afterload, worsening the regurgitant fraction and drug‐induced bradycardia further reduces cardiac output (Ko et al., 1996; Pypendop & Verstegen, 1998; Rangel et al., 2020; Vainio & Palmu, 1989).

Benzodiazepines cause minimal cardiovascular depression and can be used in association with an opioid but may cause paradoxical excitement especially in already stressed dogs (Court & Greenblatt, 1992).

Reducing systemic vascular resistances and maintaining heart rate, the off‐licence (Pypendop & Verstegen, 1998; Sarotti et al., 2024) intramuscular alfaxalone with an opioid provides a safe alternative for achieving sedation in dogs with Stage B2 MMVD (Sarotti et al., 2024). However, the large volume required might make this option less practical in large breed dogs.

#### Induction

The method of administration is more critical than the specific drug. Propofol and alfaxalone can be used safely in Stage B2 dogs when administered slowly to effect over 40 to 60 seconds (Maney et al., 2013; Sarotti et al., 2024). Co‐induction with fentanyl and/or midazolam reduces the total induction dose requirement and hemodynamic perturbations (Okushima et al., 2015). Administer propofol before midazolam to reduce excitatory effects (Sánchez et al., 2013). Ketamine use in Stage B2 remains controversial due to potential negative inotropic effects, particularly in patients with compromised sympathetic tone (Diaz et al., 1976; Pagel et al., 1992). Etomidate preserves cardiovascular stability but is costly, not widely available, and hemodynamically not much different than alfaxalone (Rodríguez et al., 2012).

#### Maintenance

While isoflurane and sevoflurane can be used, balanced anaesthetic techniques are strongly recommended to minimise volatile anaesthetic requirements and cardiovascular depression. Regional anaesthesia and/or PIVA reduce volatile anaesthetic needs (Perkowski & Oyama, 2024). Total intravenous anaesthesia (TIVA) with propofol (0.2 to 0.4 mg/kg/minute) potentially reduces hypotension compared to volatile anaesthesia (Bustamante et al., 2018; Dodam et al., 1990; Hjalmarsson et al., 2025).

#### Monitoring and support

Comprehensive anaesthetic monitoring is essential. Invasive arterial blood pressure might be needed also depending on the surgery to be performed (Bailey et al., 2025; Crosland et al., 2021). Inotropic agents (*e.g*. dobutamine) should be readily available for intraoperative hypotension given the negative inotropy of volatile anaesthetics (Goya et al., 2018). Although fluid overload must be avoided, an initial fluid rate of 3 to 4 mL/kg/hour is suggested. Further intraoperative fluid requirement should be guided by dynamic indices of fluid responsiveness when possible. In patients with Stage B2 MMVD, it is advisable to administer smaller boluses of fluid (2 to 5 mL/kg) and repeat them to effect, rather than administer large volumes (Boysen & Gommeren, 2021; Pardo et al., 2024).

#### Recovery

The recovery period is particularly critical in Stage B2 dogs. Continuous monitoring with SpO_2_, supplemental oxygen and ongoing cardiovascular assessment is essential. A stress‐free environment prevents sympathetic activation that could precipitate decompensation (Crosland et al., 2021; Höglund et al., 2012). Extended recovery monitoring is warranted given that 47% to 81% of anaesthetic deaths occur postoperatively (Brodbelt et al., 2008; Redondo et al., 2024). NSAIDs are not contraindicated for postoperative analgesia provided the patient is normotensive and normovolaemic.

### Grey zone B2 – diagnostic uncertainty anaesthetic approach

#### Clinical example

A 12‐year‐old mixed‐breed dog (8 kg) with a Grade III/VI left apical systolic murmur requires mass removal. VHS measures 11.0 and VLAS 2.5, both within grey zone ranges.

Dogs with borderline radiographic findings (VHS 10.5 to 11.5, VLAS 2.3 to 3.0) or discordant results present a common clinical challenge. These patients may represent late Stage B1 or early Stage B2, and disease stage cannot be definitively determined. Additionally, the presence of a murmur with borderline findings does not confirm MMVD, and other cardiac conditions should be considered. When additional diagnostics (NT‐proBNP, POCUS or cardiology referral) are not feasible and anaesthesia must proceed, conservative management is warranted.

The urgency of the procedure should be carefully weighed: Purely elective procedures may warrant deferring until disease stage can be clarified, while necessary procedures should proceed with enhanced precautions as outlined for Stage B2 dogs. The grey zone represents clinical reality in general practice, where practitioners frequently make anaesthetic decisions with incomplete cardiac assessment.

### Stage C and D – decompensated heart failure

Dogs with active signs of congestive heart failure (Stage C decompensated or Stage D refractory heart failure) should not undergo elective anaesthetic procedures. The presence of dyspnoea, pulmonary oedema evident on thoracic radiographs or POCUS (B‐lines), pericardial effusion (which may result from atrial tear in advanced MMVD) or other signs of decompensation represents a contraindication to routine anaesthesia until cardiac stabilisation occurs with appropriate medical management (Keene et al., 2019; Ward & DeFrancesco, 2023). Only life‐saving procedures should be considered in decompensated patients, and even then, the prognosis must be carefully discussed with owners. Cardiology consultation and referral to a specialist facility equipped for advanced monitoring and emergency intervention are strongly recommended for any dog requiring anaesthesia while in Stage C or D, as anaesthetic risk in these patients is high and detailed management is beyond the scope of this review.

### Emergency protocols: management of intraoperative complications

When anaesthetizing dogs with MMVD, careful, ongoing monitoring helps veterinarians spot problems early and respond effectively when complications develop. The sooner these problems are recognised and addressed, the better the outcome for the patient (Congdon, 2022; Perkowski & Oyama, 2024).

### Hypotension, arrhythmias and cardiovascular collapse

**FIG 6:**
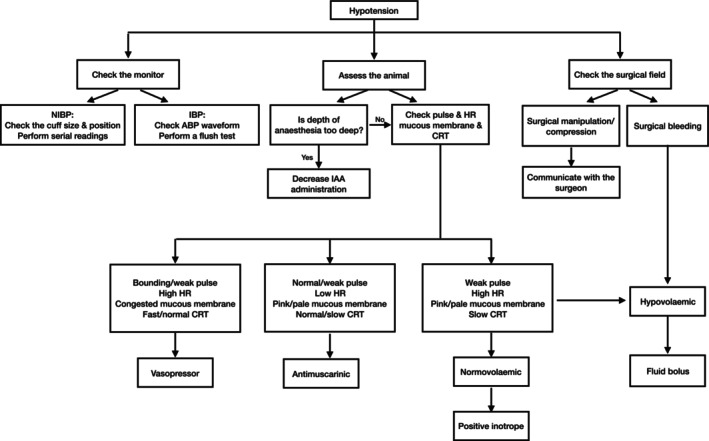
Systematic approach to troubleshooting intraoperative hypotension. The flowchart guides clinicians through assessment of monitoring equipment, patient parameters, and surgical field to identify the underlying cause of hypotension and appropriate treatment. Figure adapted from Filipas and Vettorato (2018).

Systemic blood pressure must be maintained above the minimum level required for cerebral, coronary and renal perfusion. During anaesthesia, hypotension is commonly defined as mean arterial blood pressure (MAP) <60 mmHg (Ruffato et al., 2015). However, there is neither a clear answer on the impact of intraoperative hypotension and perioperative outcome nor are there standardised guidelines for the maintenance of intraoperative blood pressure in dogs with MMVD. Unhealthy patients (ASA physical status ≥3) generally have lower blood pressure under anaesthesia than healthy patients (ASA 1 to 2) (Redondo et al., 2007). A direct approach should be used to treat hypotension by identifying and correcting the underlying cause. After ruling out potential equipment‐related issues (*e.g*. cuff position and size) and excessive anaesthetic depth, the clinician should troubleshoot hypotension in a systematic way (Fig 6) (Filipas & Vettorato, 2018).

If hypotension is associated with bradycardia (*e.g*. heart rate <60 beats/minute), the administration of an antimuscarinic drug (atropine 0.02 mg/kg or glycopyrrolate 0.01 mg/kg iv) is suggested. In dogs with MMVD, glycopyrrolate may be preferred to avoid extreme tachycardia and increased myocardial oxygen demand (Wolf et al., 2024).

If hypotension with associated to high heart rate (*e.g*. heart rate >130 to 140 beats/minute), the clinician should try to differentiate the presence of hypovolemia or hypocontractility from vasodilation. During vasodilation, mucous membranes are injected or congested with a rapid capillary refilling time (CRT); during hypovolemia and hypocontractility, mucous membranes are paler and CRT prolonged. Vasodilation can be treated administering norepinephrine to effect (0.05 to 0.5 μg/kg/minute). Acting on both α‐ and β‐adrenergic receptors, norepinephrine can increase MAP, cardiac output and oxygen delivery, primarily through vasoconstriction and improved venous return (Cannarozzo et al., 2023; Henao‐Guerrero et al., 2023; Kojima et al., 2021). If mechanical ventilation is used, dynamic indices of fluid responsiveness such as pulse pressure variation and respiratory fluctuations in aortic blood flow can help guide fluid therapy. However, the reliability of these indices is reduced in cardiopathic patients, as arrhythmias, valvular insufficiency and ventricular dysfunction can compromise the accuracy of haemodynamic predictions (Boysen & Gommeren, 2021; Fantoni et al., 2017; Gonçalves et al., 2020). In patients with MMVD, especially with pre‐anaesthetic signs of volume overload, it is advisable to administer smaller boluses of fluid (2 to 5 mL/kg) and repeat them to effect, rather than administer large quantity (Boysen & Gommeren, 2021). Inotropic agents (*e.g*. dobutamine 3 to 15 μg/kg/minute) improve myocardial contractility and are beneficial in patients with significant MR (Abdul‐Rasool et al., 1987; Goya et al., 2018; Rosati et al., 2007). Dopamine is another option to improve contractility, with dose‐dependent effects that can include inotropic and vasoconstrictive properties depending on the dose used (Abdul‐Rasool et al., 1987; Rosati et al., 2007).

Tachycardia can significantly decrease diastolic filling time, reduce cardiac output and increase myocardial oxygen demand. The most frequent cause during anaesthesia is increased sympathetic tone, due to nociception (inadequate analgesia) or inadequate anaesthesia. In those situations, hypertension is generally present and the administration of a rescue analgesic (*e.g*. fentanyl 1 μg/kg iv) or a bolus of propofol (0.5 to 1 mg/kg iv) should be considered, respectively.

Supraventricular arrhythmias (typically characterised by narrow QRS complexes not preceded by sinus P waves) or ventricular arrhythmias (characterised by wide, bizarre‐looking QRS complexes with AV dissociation, not preceded by sinus P waves) may occur in dogs with MMVD, especially in stages C‐D (Crosara et al., 2010; Franchini et al., 2021; Guglielmini et al., 2020). In the presence of tachyarrhythmias, antiarrhythmics should be administered as necessary before the anaesthetic for stabilisation. For example, in the presence of atrial fibrillation, diltiazem with digoxin is generally started preoperatively to obtain optimal control of heart rate (targeting a mean heart rate ≤125 bpm) (Keene et al., 2019; Pedro et al., 2023; Romito, Bertarello, et al., 2025).

In pre‐operative planning, dogs affected by haemodynamically significant ventricular tachyarrhythmias might be treated with sotalol or amiodarone (Romito et al., 2024) and, if refractory, adding mexiletine (Romito, Mazzoldi, et al., 2025). The effect of these antiarrhythmic drugs on the cardiovascular system and the interaction with the commonly used anaesthetic drugs need to be considered. For example, in dogs treated with digoxin, hypokalaemia should be prevented, as it may increase the risk of digitalis toxicity (Kawamura et al., 1978; Steiness, 1978). Therefore, potassium levels should be checked prior to anaesthesia and corrected as needed. Moreover, intravenous sodium channel blockers (*i.e*. lidocaine) may have negative vasodilatory effects if rapidly injected (Edouard et al., 1986; Güler et al., 2023) and beta blockers (*i.e*. esmolol and atenolol) and calcium channel blockers (*i.e*. diltiazem and verapamil) may have negative inotropic and vasodilatory effects (Miyamoto et al., 2000, 2001; Verschoor‐Kirss et al., 2022). Therefore, balanced anaesthetic techniques aiming to reduce the use of inhalational anaesthetic agents should be pursued. At the same time, the administration of positive inotropes (*i.e*. dobutamine), antimuscarinic and vasoactive drugs (*i.e*. norepinephrine) might be needed to improve cardiac output and blood pressure (Perkowski & Oyama, 2024).

When arrhythmias occur during anaesthesia, initial assessment should verify heart rate consistency across ECG, pulse oximetry, manual pulse counting and arterial blood pressure.

For ventricular tachycardia causing hypotension, lidocaine (1 to 2 mg/kg iv bolus, followed by 1.5 to 6 mg/kg/hour CRI if needed) is preferred, given its high efficacy and low rate of side effects in dogs (Bruchim et al., 2012; Seo et al., 2022). In cases of ventricular tachyarrhythmias refractory to lidocaine, intravenous alternatives include esmolol (Verschoor‐Kirss et al., 2022) and premixed amiodarone (Levy et al., 2016). For supraventricular tachycardia, vagal manoeuvres or intravenous administration of diltiazem (Miyamoto et al., 2000, 2001) or esmolol (Verschoor‐Kirss et al., 2022) can be utilised. Moreover, in the rare case of acute vagally mediated rhythm disturbances that may develop during anaesthesia, such as atrial fibrillation and atrial dissociation, intravenous lidocaine represents the first‐line treatment option (Moïse et al., 2005; Romito et al., 2022). For life‐threatening ventricular fibrillation or pulseless ventricular tachycardia, immediate chest compressions and defibrillation are required (Rozanski et al., 2012).

Severe bradyarrhythmias, including high‐grade atrioventricular blocks, may also occur secondary to degenerative changes in the conduction system (Kaneshige et al., 2007). In such cases, permanent pacing may be required (Santilli et al., 2019).

For cardiovascular collapse or cardiac arrest, epinephrine (0.01 mg/kg bolus) may be administered and cardiopulmonary resuscitation performed according to the most recent RECOVER guidelines (Burkitt‐Creedon et al., 2024; Grimm et al., 2015; Macintire et al., 2017; Marotto et al., 2018; Pascoe et al., 2006; Perkowski & Oyama, 2024; Wolf et al., 2024). Detailed drug dosages are provided in Table 2.

**Table 2:** Anaesthetic protocol and emergency management for dogs with MMVD

Category	Agent	Dose/rate	Considerations
Premedication			
Opioids	Hydromorphone Morphine Methadone Butorphanol Buprenorphine Fentanyl	0.05 to 0.2 mg/kg iv, im, sc 0.1 to 0.5 mg/kg im, sc 0.1 to 0.3 iv, im, sc 0.1 to 0.4 mg/kg iv, im, sc 10 to 30 μg/kg iv, im, sc 5 to 7 μg/kg iv	Emesis Emesis. IV might cause histamine release Use as co‐inductor. May cause bradycardia
Benzodiazepines	Midazolam Diazepam	0.2 to 0.5 mg/kg iv, im 0.2 to 0.5 mg/kg iv	Minimal cardiovascular depression but excitation is likely
Phenothiazines	Acepromazine	10 to 50 μg/kg iv, im, sc	Risk of vasodilation and hypotension
α2‐agonists	Dexmedetomidine Medetomidine + Vatinoxan	0.5 to 1 μg/kg iv 2 to 3 μg/kg im (medetomidine 5 to 10 μg/kg) + vatinoxan 100 to 200 μg/kg im	Could be used in B1 MMVD but bradycardia is likely Could be used to sedate B1 MMVD not undergoing anaesthesia
Neurosteroid	Alfaxalone	1 to 2 mg/kg im	Large volume. Do not use alone
Induction			
Alkylphenol	Propofol	2 to 6 mg/kg iv to effect	Slow administration (60 seconds). Possible transient hypotension
Carboxylated imidazole derivative	Etomidate	0.5 to 2 mg/kg iv to effect	Poor induction quality. Do not use alone.
Neurosteroid	Alfaxalone	1 to 3 mg/kg iv to effect	Slow administration (60 seconds). Possible increase HR
Maintenance			
Volatile anaesthetic	Isoflurane Sevoflurane	MAC in dogs 1.2% to 1.3% MAC in dogs 2.3% to 2.4%	Dose‐dependent vasodilation and decrease contractility. Use regional anaesthesia or PIVA to reduce MAC
Total intravenous anaesthesia (TIVA)	Propofol infusion	0.2 to 0.4 mg/kg/minute	Less hypotension than volatile anaesthetic. Use regional anaesthesia or PIVA to decrease total dose
Intraoperative analgesia			
Regional anaesthesia	Lidocaine Bupivacaine	1 to 4 mg/kg 1 to 3 mg/kg	Dose and dilution depend on the type of block performed
Partial intravenous anaesthesia (PIVA)	Fentanyl Ketamine Lidocaine	5 to 10 μg/kg/hour iv 0.3 to 0.6 mg/kg/hour iv 1 to 2 mg/kg/hour iv	Bradycardia and hypoventilation Possible vasodilation and decreased contractility
Blood pressure and arrhythmias management			
Positive inotropes	Dobutamine Dopamine Norepinephrine	3 to 15 μg/kg/minute iv 3 to 7.5 μg/kg/minute iv 0.05 to 0.5 μg/kg/minute iv	Improves contractility and reduces afterload Improves contractility. At higher doses might cause vasoconstriction Improves contractility and cause dose‐dependent vasoconstriction
Antimuscarinic	Atropine Glycopyrrolate	0.02 to 0.04 mg/kg iv 5 to 10 μg/kg iv	To treat bradycardia and bradyarrhythmias. Atropine can cause more tachyarrhythmias
Na^+^ channel blocker	Lidocaine	2 to 4 mg/kg iv (up to 8 mg/kg within 30 minutes) followed by 1.5 to 5 mg/kg/hour iv	To treat ventricular tachycardia and vagally associated atrial fibrillation and atrial dissociation
Ca^2+^ channel blocker	Diltiazem	0.05 to 0.15 mg/kg iv over 5 minutes; additional boluses as needed q 15 minutes up to 0.5 mg/kg cumulative dose	To treat supraventricular tachycardia. Monitor for bradycardia and hypotension
Beta blocker	Esmolol	0.05 to 0.5 mg/kg iv over 2 to 5 minutes; then 10 to 200 μg/kg/minute if needed	To treat supraventricular tachycardia and ventricular tachycardia refractory to lidocaine. Monitor for bradycardia and hypotension
Class III antiarrhythmic	Amiodarone (premixed)	2 mg/kg iv bolus over 10 minutes, followed by CRI of 0.8 mg/kg/hour for 6 hours and 0.4 mg/kg/hour thereafter	To treat ventricular tachycardia refractory to lidocaine
Defibrillation	Biphasic Monophasic	2 to 4 J/kg 4 to 6 J/kg	To treat ventricular fibrillation or pulseless ventricular tachycardia. Increase by 50% if unsuccessful
Alpha–beta agonist	Epinephrine	0.01 mg/kg iv bolus every 3 to 5 minutes	Cardiac arrest with non‐shockable rhythms

Drug selection should prioritise maintenance of forward cardiac output, afterload reduction, avoidance of excessive bradycardia and maintenance of contractility. Dosages are based on published literature, clinical guidelines and clinical experience; adjust based on individual patient assessment

HR Heart rate, im Intramuscular, iv Intravenous, MAC Minimum alveolar concentration, MMVD Myxomatous mitral valve disease, PIVA Partial intravenous anaesthesia, sc Subcutaneous, TIVA Total intravenous anaesthesia

This review provides a practical framework for anaesthetic risk assessment in dogs with MMVD. The ASA physical status classification proves inadequate for dogs with MMVD, particularly given substantial heterogeneity within preclinical stages.

General practitioners have accessible tools for pre‐anaesthetic cardiac assessment including physical examination, radiographic measurements (VHS/VLAS), cardiac biomarkers (NT‐proBNP) and POCUS. This framework provides guidance for safe anaesthetic management from confirmed Stage B1 through grey zone cases to Stage B2, recognising that while specialist evaluation remains ideal, practical alternatives can guide safe anaesthetic management for this commonly encountered cardiac condition.

### Author contributions

**I. Levinzon:** Conceptualization; investigation; funding acquisition; writing – original draft; methodology; visualization; writing – review and editing; project administration. **L. Köster:** Conceptualization; investigation; writing – original draft; methodology; visualization; writing – review and editing; supervision. **E. Vettorato:** Conceptualization; investigation; writing – original draft; methodology; visualization; writing – review and editing; supervision. **A. H. Estrada:** Conceptualization; investigation; writing – original draft; funding acquisition; methodology; visualization; writing – review and editing; project administration; supervision.

### Conflict of interest

The authors have no conflicts of interest to declare.

## Funding

This work was supported by the Stanton Foundation.

## Data Availability

The data that support the findings of this study are available in PubMed/MEDLINE at https://pubmed.ncbi.nlm.nih.gov/.
